# Topographic analysis of local OCT biomarkers which predict progression to atrophy in age-related macular degeneration

**DOI:** 10.1007/s00417-024-06389-x

**Published:** 2024-02-01

**Authors:** Navid Manafi, Alireza Mahmoudi, Mehdi Emamverdi, Giulia Corradetti, Stephanie Trejo Corona, Charles C. Wykoff, SriniVas R. Sadda

**Affiliations:** 1 Doheny Image Reading and Research Laboratory, Doheny Eye Institute, 150 N. Orange Grove Blvd, Suite 232, Pasadena, CA 91103 USA; 2 Department of Ophthalmology, David Geffen School of Medicine, University of California-Los Angeles, Los Angeles, CA USA; 3 Retina Consultants of Texas, Retina Consultants of America, Houston, TX USA

**Keywords:** AMD, cRORA, IHRF, OCT, Retinal atrophy, Age-related macular degeneration

## Abstract

**Purpose:**

To define optical coherence tomography (OCT) biomarkers that precede the development of complete retinal pigment epithelium and outer retinal atrophy (cRORA) at that location in eyes with age-related macular degeneration (AMD).

**Methods:**

In this retrospective case–control study, patients with dry AMD who had evidence of cRORA and OCT data available for 4 years (48 ± 4 months) prior to the first visit with evidence of cRORA were included. The visit 4 years prior to the development of cRORA was defined as the baseline visit, and the region on the OCT B-scans of future cRORA development was termed the case region. A region in the same eye at the same distance from the foveal center as the case region that did not progress to cRORA was selected as the control region. OCT B-scans at the baseline visit through both the case and control regions were evaluated for the presence of soft and cuticular drusen, drusen with hyporeflective cores (hcD), drusenoid pigment epithelial detachments (PED), subretinal drusenoid deposits (SDD), thick and thin double-layer signs (DLS), intraretinal hyperreflective foci (IHRF), and acquired vitelliform lesions (AVL).

**Results:**

A total of 57 eyes of 41 patients with dry AMD and evidence of cRORA were included. Mean time from the baseline visit to the first visit with cRORA was 44.7 $\pm$ 6.5 months. The presence of soft drusen, drusenoid PED, AVL, thin DLS, and IHRF at the baseline visit was all associated with a significantly increased risk of cRORA at that location. Multivariable logistic regression revealed that IHRF (OR, 8.559; *p* < 0.001), drusenoid PED (OR, 7.148; *p* = 0.001), and a thin DLS (OR, 3.483; *p* = 0.021) were independent predictors of development of cRORA at that location.

**Conclusions:**

IHRF, drusenoid PED, and thin DLS are all local risk factors for the development of cRORA at that same location. These findings would support the inclusion of these features within a more granular staging system defining specific steps in the progression from early AMD to atrophy.

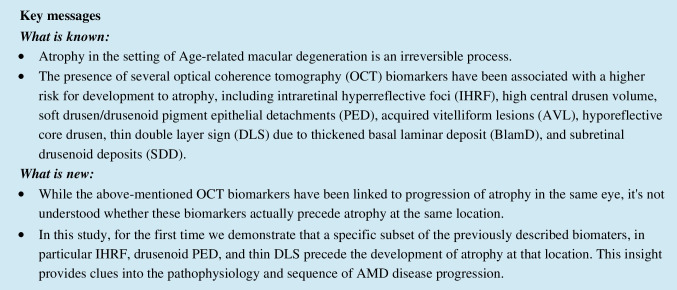

## Introduction

With a prevalence of 196 million cases in 2020 and with the projected prevalence to rise to 288 million by 2040, age-related macular degeneration (AMD) is the most common cause of blindness in developed countries and accounts for 8.7% of blindness worldwide [1, 2]. Geographic atrophy (GA) and macular neovascularization (MNV) represent the late stage of AMD and are not mutually exclusive. GA has classically been defined on color photographs, but more recently the Classification of Atrophy Meetings (CAM) group defined the characteristics of atrophy on OCT and introduced the term complete retinal pigment epithelium and outer retinal atrophy (cRORA) [3].

In February of 2023, a treatment for GA, pegcetacoplan, was cleared by the FDA [4]. The treatment moderately slows the progression of GA and does not reverse the areas of atrophy and visual loss that has already occurred. Thus, there has been significant interest in developing treatments targeting earlier stages of the disease process.

In order to define earlier potential intervention points for use in clinical trials, there has been an intense focus to identify biomarkers that may increase the risk for development of GA. A number of such biomarkers have been established on OCT including intraretinal hyperreflective foci [5], high central drusen volume, soft drusen/drusenoid pigment epithelial detachments (PED) [6], acquired vitelliform lesions [7], hyporeflective core drusen [8], thin double-layer sign due to thickened basal laminar deposit [9], and subretinal drusenoid deposits [10].

These biomarkers have largely been studied in eye-level analyses, meaning that the presence of the biomarker in an eye increases the risk of GA development in that eye, but not necessarily at the location of the biomarker. If one were to establish a new staging system to describe the progression of AMD that could be used to support future clinical trials, however, it would be important to know if these high-risk biomarkers actually predispose for the development of the cRORA at that same topographic location. In other words, it would be helpful to determine if these biomarkers are actually precursors for the development of cRORA. This information would both support the development of a new OCT-based staging system and also provide new insights into the pathophysiologic mechanisms and sequence of events leading to the development of GA [11].

Thus, in this study, we performed a lesion-level analysis to define which OCT biomarkers were associated with increased risk for the development of cRORA at that location using a case–control approach within the same eye.

## Methods

### Overall design and inclusion and exclusion criteria

In this retrospective cohort study, patients with non-neovascular AMD who had evidence of cRORA and OCT data available for 4 years (48 ± 4 months) prior to the first visit with evidence of cRORA were included. Patients who met these criteria were selected from among patients examined at Retina Consultants of Texas (Houston, TX, USA) between 2015 and 2022. The study adhered to the tenets of the Declaration of Helsinki and was approved by the institutional Review Boards (IRB) of the Houston Methodist Hospital (Pro00020661:1 “Retrospective Prospective Analysis of Retinal Diseases”) and the University of California Los Angeles (IRB#15–000083—Ocular Imaging Study). As the data collection was retrospective, a waiver of informed consent was granted.

Eyes that had evidence of macular neovascularization (MNV), vitreoretinal surgery, co-existence of any retinal diseases causing significant abnormalities of the retinal layers (including severe epi-retinal membrane (ERM) or diabetic retinopathy (DR)), and/or intravitreal injection during or before this 48-month study period were excluded.

### Image acquisition and grading procedure

All OCTs at baseline and follow-up visits were obtained using a Spectralis OCT (SPECTRALIS; Heidelberg Engineering, Inc., Heidelberg, Germany) with a volume scanning protocol of 49 B-scans over 6 × 6 mm with an automatic real time of 6 (i.e., 6X averaging) and with the use of the follow-up/reference scan function on the device to allow more precise alignment of scans between visits. The records and longitudinal images for all subjects with AMD were reviewed to identify the first visit at which cRORA was present on OCT. The visit 48 months before the development of cRORA was defined as the baseline visit for this analysis.

At the baseline visit, all OCT A-scans at the location of future cRORA, defined as the case region within the eye, were evaluated for the presence of soft drusen, drusenoid pigment epithelial detachments [6], drusen with hyporeflective cores (hcD), subretinal drusenoid deposits (SDD), cuticular drusen, thick and thin double-layer signs (DLS), intraretinal hyperreflective foci (IHRF), and acquired vitelliform lesions (AVL). A region within the same eye at the same distance from the fovea as the region which progressed to cRORA that did not progress to cRORA over 48 months was defined as the control region. The control region was selected in close proximity to the case region, but did not overlap with the case region. For eyes in which the cRORA developed at a subfoveal location, it was not possible to select an equidistant control region, but the control region was again selected as close as possible to the case region. Distance to the fovea was chosen a key parameter for matching case and control regions as it has been shown that the choriocapillaris flow deficit increases with proximity to the fovea [12, 13], and the status of the choriocapillaris is thought to be an important element in AMD progression [14–16].

Qualitative and quantitative assessment and gradings were performed by two certified, independent masked Doheny Image Reading and Research Lab (DIRRL) graders (NM and AM). For cases in which the graders had uncertainty or had discrepant grades, the DIRRL medical director (SS) provided the final determination. Differences between graders were also assessed to determine the reproducibility of the grading.

### Definitions of lesions and biomarkers

**Fig. 1:**
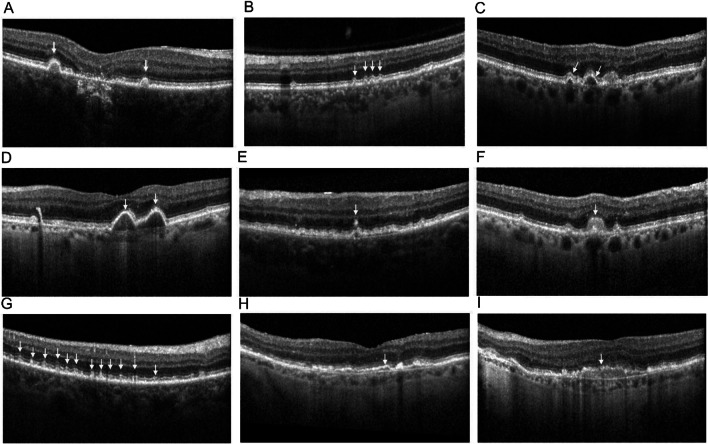
Figure examples of biomarkers evaluated 4 years prior to development of complete retinal pigment epithelium and outer retina atrophy (cRORA). **A** Soft drusen. **B** Cuticular drusen. **C** Hyporeflective core drusen (hcD). **D** Drusenoid pigment epithelial detachment (PED). **E** Intraretinal hyperreflective foci (IHRF). **F** Acquired vitelliform lesion (AVL). **G** Subretinal drusenoid deposits (SDD). **H** Thin double-layer sign (DLS). **I** Thick DLS

**Fig. 2:**
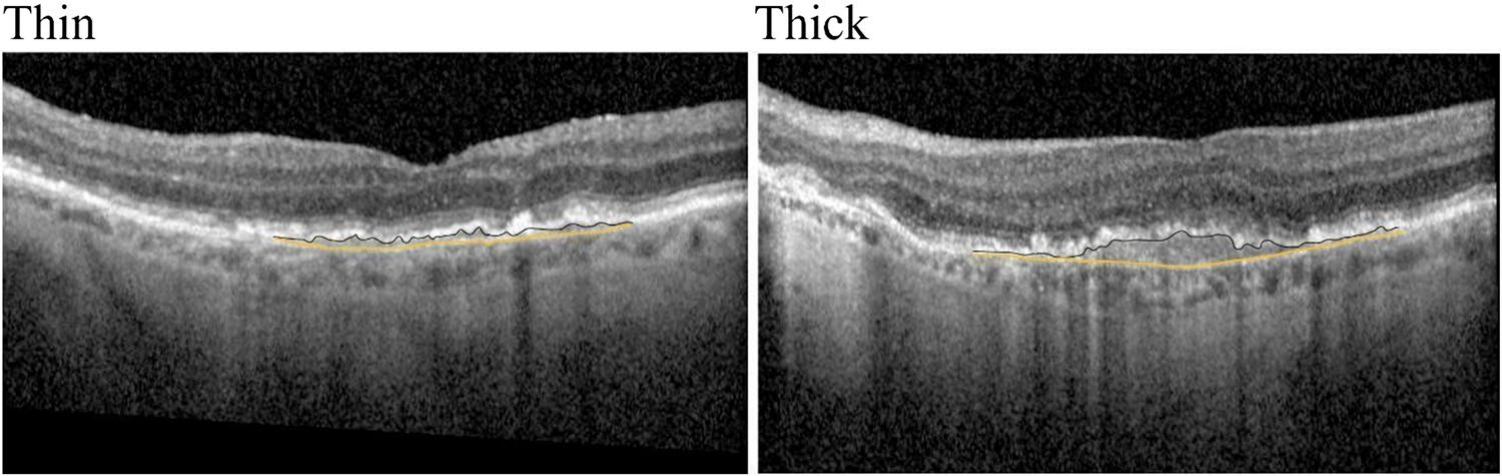
The thin and thick double-layer sign (DLS). The yellow and black markings represent the inner margin of Bruch’s membrane and outer margin of retinal pigment epithelium (RPE), respectively

The following definitions of biomarkers from the DIRRL central grading protocol were utilized in this study:

The term cRORA was defined as any lesion with outer retinal and RPE layer atrophy with hyper-transmission defect of equal to or more than 250 μm [17]. IHRF were defined as lesions at least 3 pixels in size within the neurosensory retina with a reflectivity equal to or greater than RPE [18]. Drusen and drusenoid PED were detected as elevations of the RPE with a smooth contour and moderate to high homogenous internal reflectivity. The lesion was classified as a drusen if the basal width was > 63 µm and < 350 µm and as a drusenoid PED if the greatest basal width was ≥ 350 µm [6, 19]. AVL was identified as a dome-shaped hyperreflective area bordered posteriorly by the inner border of the RPE and anteriorly by the ellipsoid zone, ELM, or outer border of the ONL [7]. DLS was identified by the presence of an irregular area of RPE elevation with a clear separation between the RPE and Bruch’s membrane. In accordance with previous reports [9], DLS was classified as a thin DLS if only a single zone of low to medium reflectivity occupied the region between the Bruch membrane and the RPE and as a thick DLS if multiple layers with different reflectivity could be discerned [9]. SDD was defined as accumulation of small mound or cone-shaped deposits between the RPE and photoreceptors. At least three SDD lesions had to be identified in order for the diagnosis to be made (Figs. 1 and 2) [20].

### Statistical analysis

All statistical analyses were performed using the SPSS software (IBM Corp. Released 2021. IBM SPSS Statistics for Macintosh, Version 28.0. Armonk, NY: IBM Corp). For qualitative variables, cross-tabulation (crosstab) was used to assess the relationship between variables. A chi-square test was used to assess the relationship between categorical variables. For quantitative analysis, first the dataset was assessed for the distribution of data using the Kolmogorov–Smirnov test (KS test). As the distribution of data was not normal, the Mann–Whitney *U* test was used to compare differences between the two groups.

For the assessment of the strength of associations, trend analysis, and predictive power of the different variables, a regression analysis was performed. Univariate regression was initially performed and parameters which had a *p*-value of < 0.1 from the univariate analysis were then included in the multiple logistic regression analysis to identify independent significant factors. Odds ratios (ORs) were computed. For the assessment of agreement between graders, Cohen’s kappa (*κ*) coefficient was calculated. The level of significance was set at 0.05 and *p*-values below 0.05 were considered statistically significant.

## Results

A total of 57 eyes of 41 patients met the inclusion criteria and were included in this study. The mean age of the patients was 74.5 ± 8.4 years. The cohort consisted of 25 (66%) females and 13 (34%) males. The mean duration of follow-up between the baseline assessment and the first visit with cRORA was 44.5 ± 6.4 months (range, 36–52 months).

A comparison of the frequency of features present at the baseline visit in the case and control regions is shown in Table 1. With the exception of SDD, all of the studied biomarkers were found more frequently in the case regions (i.e., those regions that progressed to cRORA) compared to the control regions, though the difference was only statistically significant for IHRF, drusenoid PED, soft drusen, thin DLS, and AVL. Out of 57 eyes, only three eyes had subfoveal lesions.

**Table 1:** The frequencies of different biomarkers in the control and case regions at baseline. *PED*, pigment epithelial detachments; *SDD*, subretinal drusenoid deposits; *DLS*, double-layer sign; *AVL*, acquired vitelliform lesions; *IHRF*, intraretinal hyperreflective foci; *hcD*, drusen with hyporeflective core

	Controls	Cases	*p*-value
Soft drusen	17 (29.8%)	27 (47.4%)	**.041**
hcD	3 (5.3%)	8 (14.0%)	.102
Cuticular drusen	9 (15.8%)	16 (28.1%)	.087
Drusenoid PED	5 (8.8%)	21 (36.8%)	** < .001**
SDD	2 (3.5%)	0 (0.0%)	.248
AVL	2 (3.5%)	8 (14.3%)	**.044**
Thin DLS	9 (15.8%)	21 (37.5%)	**.008**
Thick DLS	2 (3.5%)	3 (5.3%)	.500
IHRF	4 (7.0%)	27 (47.4%)	** < .001**

**Fig. 3:**
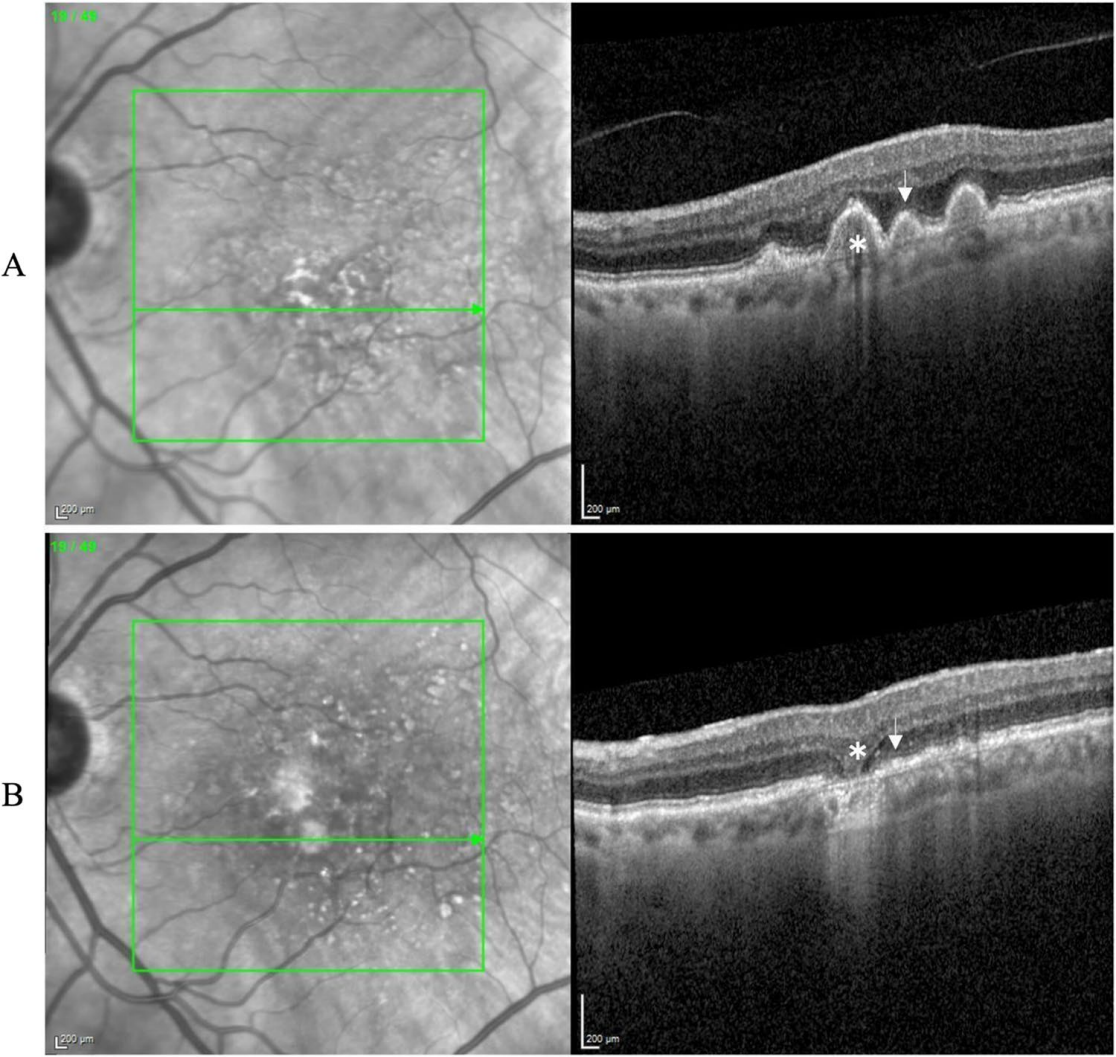
En-face (left) and B-scan (right) at the baseline (**A**) and 4-year follow-up visit (**B**). The “case” area is represented by an asterisk and the “control” adjacent to it is represented by an arrow. The case area had a large drusen and overlying IHRF 4 years before development of cRORA, while the control area had a smaller drusen without IHRF in this patient. cRORA complete retinal pigment epithelial and outer retinal atrophy, IHRF intraretinal hyperreflective foci. The Spectralis OCT was used

**Fig. 4:**
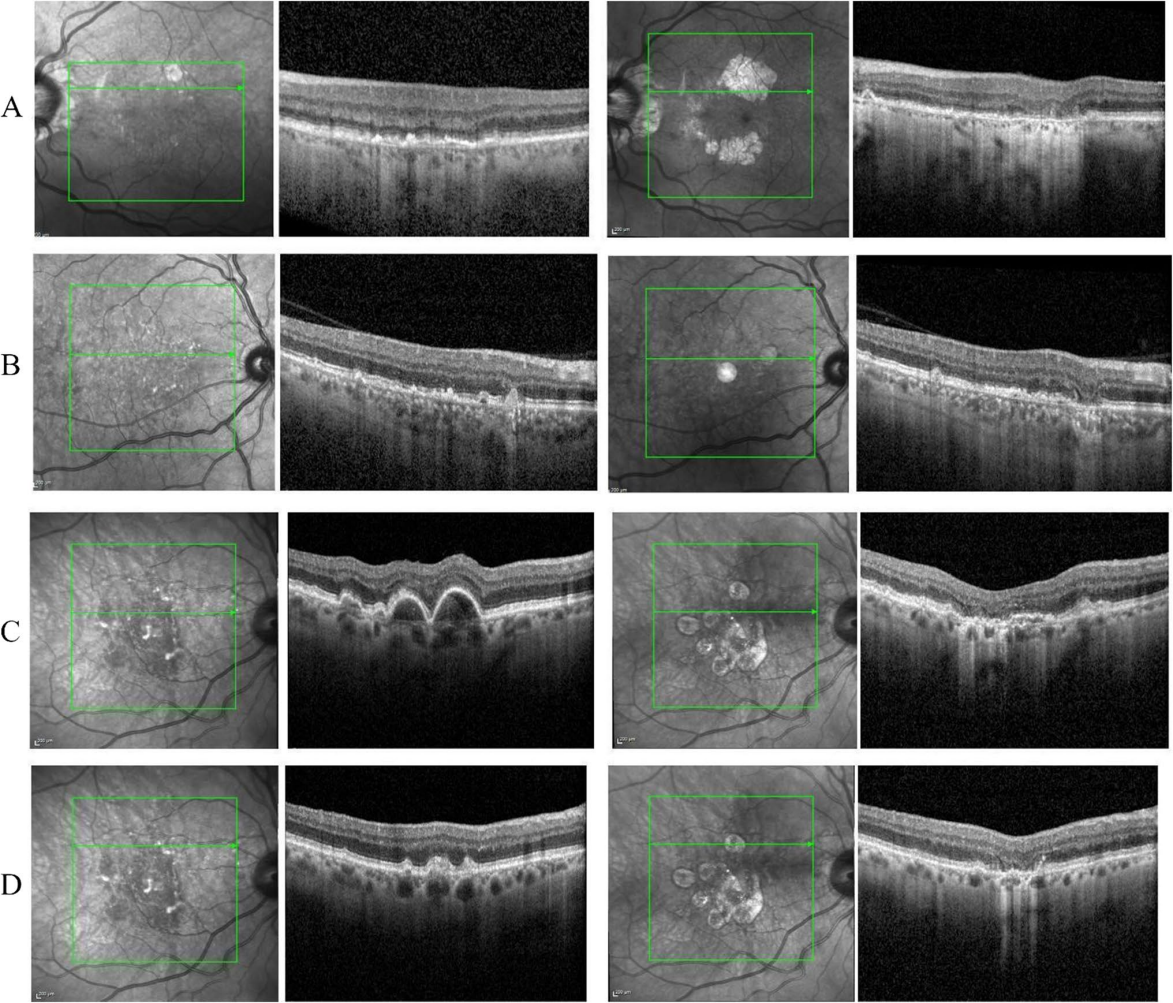
Progression to complete RPE and outer retinal atrophy (cRORA) in several eyes with dry age-related macular degeneration (AMD): Figure **A** belongs to the left eye of a patient with thin double-layer sign at baseline (left) and progression to atrophy within 4 years at the same location (right). Figure **B** belongs to the right eye of a patient with soft drusen at baseline (left) and progression to atrophy in four years (right). Figures **C** and **D** belong to the right eye of a patient with pigment epithelial detachment (PED) and acquired vitelliform lesion (AVL) at baseline, respectively; the corresponding images on the right for **C** and **D** depict the presence of atrophy in the same location. For each case, both the en-face and B-scan view have been presented for comparison

Logistic regression analysis was performed to assess relative strength of the different biomarkers as risk factors for progression to cRORA at that location (Table 2). The multivariable analysis revealed that IHRF, drusenoid PED, and a thin DLS were independent risk factors for progression to cRORA at that location with odds ratios for progression of 9.273 (*p* < 0.001), 7.065 (*p* = 0.001), and 3.597 (*p* = 0.017), respectively (Figs.3 and 4).

**Table 2:** Results of the logistic regression analysis of the biomarkers. *OR*, odds ratio; *CI*, confidence interval; *PED*, pigment epithelial detachments; *SDD*, subretinal drusenoid deposits; *DLS*, double-layer sign; *AVL*, acquired vitelliform lesions; *IHRF*, intraretinal hyperreflective foci; *hcD*, drusen with hyporeflective core

Baseline variables	Univariate analysis (logistic regression)			Multiple logistic regression		
	Odds ratios	95% CI	*p*-value	Odds ratios	95% CI	*p*-value
Drusenoid PED	6.067	2.094–17.578	< .001	7.065	2.148–23.239	**.001**
Thin DLS	3.200	1.309–7.825	.011	3.597	1.255–10.311	**.017**
IHRF	11.925	3.808–37.345	< .001	9.273	2.778–30.956	** < .001**
Soft drusen	2.118	.981–4.572	.056	1.205	.417–3.483	.730
AVL	4.583	.928–22.636	.062	3.556	.582–21.727	.170
hcD	2.939	.738–11.706	.126			
Cuticular drusen	2.081	.832–5.206	.117			
Thick DLS	1.528	.246–9.507	.650

## Discussion

In this retrospective case–control study, we aimed to identify specific OCT biomarkers or precursor lesions which increased the risk of progression to cRORA within 4 years at that precise location. We observed that several lesions including IHRF, drusenoid PED, soft drusen, thin DLS, and AVL were commonly present at these locations that eventually progressed to cRORA, with IHRF, drusenoid PED, and thin DLS manifesting as independent predictors.

The most important or strongest predictor or precursor lesion in our study was IHRF. It should be noted that IHRF in eyes with AMD have been suggested to arise from multiple sources including extravasated lipoproteins [21] and activated microglia [22], as well as dissociated RPE cells that have migrated intraretinally [23]. Dissociated RPE cells are one of many phenotypes of distressed RPE cells described by Curcio and colleagues [24]. Given that they are a sign of distressed RPE, it is perhaps not surprising that cRORA frequently ensues in this location.

It is notable that IHRF appeared to be a substantially stronger risk factor for local progression to atrophy compared to drusenoid PED. This might suggest that IHRF are a later phenotype/precursor or feature that appears more proximal to the time of development of atrophy compared to a drusenoid PED [25]. Recently, Au et al. demonstrated that eyes with taller drusen or drusenoid PED tended to demonstrate other high features for progression to atrophy including IHRF [26]. In particular, IHRF were most commonly observed over the apex of these large drusen or drusenoid PED. This observation led to the formulation of a pathophysiologic hypothesis regarding the development of large drusen and the eventual appearance of IHRF, which appears to be corroborated by histopathologic studies [24]. Specifically, the pathologic process may start with oxidative injury and structural and metabolic alterations of RPE cells, through a combination of various factors, including aging, diet, and other environmental exposures (e.g., smoking, sunlight).

The RPE cell injury and metabolic impairment may also have secondary impacts on adjacent photoreceptors and choriocapillaris, ultimately resulting in the accumulation of extracellular excretory materials beneath the RPE cells in Bruch’s membrane, giving rise to drusen [27, 28]. The progressive accumulation of these materials and enlargement of drusen increases the separation between the RPE cells and the underlying choriocapillaris, exacerbating RPE ischemia and accentuating the RPE injury and dysfunction. This effectively generates a vicious cycle of progressive RPE distress which can trigger the “activation” of these RPE cells. One of these activation phenotypes, as already noted, is the dissociation of RPE cells from the monolayer, which can allow these cells to migrate intraretinally, giving rise to IHRF. We further theorize that these cells may be attracted by the nutrient supply available in the overlying retinal deep capillary plexus (DCP)—which may be physically closer to the RPE cells than the choriocapillaris in the setting of tall drusen. The fact that the IHRF appear specifically at the apex of these drusen [26] further supports the concept of the importance of the distance between the RPE and the choriocapillaris. We should note, however, that the pathophysiology of AMD is not yet fully elucidated and it remains uncertain whether the RPE, choriocapillaris, photoreceptors, or Bruch’s membrane is the primary site of disease and whether this varies from patient to patient.

In addition to drusenoid PED and IHRF, a thin DLS, a feature thought to correspond to regions of thickened basal laminar deposit, was shown to be independently associated with progression to atrophy in an eye-level analysis [9, 29]. In the present study, a thin DLS was also a risk factor for progression at the lesion level. The separation between the RPE and choriocapillaris is relatively small in these regions, which would appear to be inconsistent with pathophysiologic hypothesis related to tall drusen and the importance of physical separation between the RPE and the choriocapillaris. We would hypothesize that the choriocapillaris may be more severely impaired in regions with thickened basal laminar deposit (i.e., thin DLS) and may account for why atrophy tends to appear in these regions. This hypothesis, however, needs to be evaluated in future OCT angiography studies.

AVL were another potential cRORA precursor that was evaluated in our study. The primary site of pathology for AVL is thought to be the RPE, where the accumulation of vitelliform material is thought to occur due to a decrease in the RPE’s ability to clear photoreceptor outer segments [30, 31]. Histopathological studies reveal a significant accumulation of macrophages in the subretinal space which highlights the role of inflammation which may be relevant to the clearance of the vitelliform material and subsequent development of atrophy [32–34]. AVL, however, did not remain independent risk factors for progression to atrophy in our analysis. It should be noted, however, that we had relatively few AVL in our cohort, and thus, we may have been underpowered to demonstrate the risk associated with these lesions. Alternatively, many eyes with AVL can develop IHRF prior to the collapse of these lesions, and the collinearity with IHRF may also prevent AVL from appearing as independent risk features.

SDD did not present as important local risk factors in our analysis, but very few eyes in our series had SDD. This low prevalence of SDD in our cohort is likely because our analysis was limited to the 6 × 6 mm macular OCT region, whereas SDD tend be more prevalent along the arcades [35]. Patients with SDD tend to have thinner choroids, more severe choriocapillaris impairment, a higher risk for development of type 3 MNV, and a higher risk for atrophy [10]. Atrophy tends to develop centrally in these patients despite the greater prevalence of SDD outside of the macula [36]. This could also account for why SDD themselves may not be a predictor for atrophy at that same location. SDD are thought to develop in rod-rich regions which may explain their prevalence outside the macula. The choriocapillaris is diffusely impaired in these eyes with SDD, but the choriocapillaris is generally more severely impaired in the central macula which may explain the central development of atrophy in these eyes despite the extramacular location of SDD [37].

Our study had a number of limitations which should be considered when assessing our results. Most notably, the study was retrospective and thus subject to ascertainment bias and unknown confounders. We tried to mitigate against ocular or systemic confounders by choosing control regions within the same eye. Moreover, as the choriocapillaris is known to have a regional dependence, we further mitigated a potential confounder by choosing the control region at a similar distance from the foveal center as the case region. This approach, however, does not mitigate against differences that may exist between the superior vs. inferior or temporal vs. nasal retina. Second, the OCT scan protocol has an inter-scan spacing of ~ 120 microns. It is possible that there were features that were missed between the B-scans that may have been relevant to the development of atrophy in this region. Third, we only considered a set of pre-defined features for evaluation at the baseline visit. It is possible that there were other abnormalities present at these locations that were not graded that were important predictors for progression. A post hoc review, however, did not disclose other findings or abnormalities in these regions. Fourth, while we selected control regions in close proximity to the case region, it is possible that there was an unknown bias in the selection of these regions that we cannot account for. Furthermore, for cases with subfoveal cRORA, the control region could not be selected at a precisely equidistant location relative to the fovea. However, there were only three cases with subfoveal lesions, and we are doubtful this has any significant impact on our findings. Another limitation is the relatively small sample size and the low frequency of certain features such as AVL and SDD, which left the analysis underpowered to detect small effects. In addition, we did not include drusen volume and height in our analyses, and they may have impacted the fate of drusen. Moreover, because drusen height was not considered, we could not specifically test our hypothesis that increasing distance between the RPE and choriocapillaris is an important aspect of the pathophysiology of progression to atrophy. This concept, however, is being evaluated in a separate study. A final limitation is that our findings are specific to the 4-year interval of our study. Other risk factors may prove to be important if shorter or longer intervals are chosen.

Despite these limitations, our study has several strengths including its case–control design, long follow-up interval to assess for development of atrophy, and the use of certified reading center graders with a standardized grading protocol.

In summary, in patients’ eyes with early or intermediate AMD, the presence of IHRF, drusenoid PED, and a thin DLS appears to increase the risk of development of cRORA at that location over 4 years. As these lesions appear to be important precursors for the development of atrophy, they may be useful for informing a more granular OCT-based staging system and for defining new endpoints for potential future early therapeutic intervention.
